# CMV-Specific Cell-Mediated Immunity in Immunocompetent Adults with Primary CMV Infection: A Case Series and Review of the Literature

**DOI:** 10.3390/v13050816

**Published:** 2021-05-01

**Authors:** Angela Chiereghin, Gabriella Verucchi, Tiziana Lazzarotto

**Affiliations:** 1 Section of Microbiology, Department of Specialized, Experimental and Diagnostic Medicine, University of Bologna, 40138 Bologna, Italy; angela.chiereghin2@unibo.it; 2 Department of Public Health, Local Health Authority of Bologna, 40121 Bologna, Italy; 3 Infectious Diseases Unit, Department of Medical and Surgical Sciences, IRCCS St. Orsola Polyclinic, University of Bologna, 40138 Bologna, Italy; gabriella.verucchi@unibo.it; 4 Microbiology Unit, Department of Specialized, Experimental and Diagnostic Medicine, IRCCS St. Orsola Polyclinic, University of Bologna, 40138 Bologna, Italy

**Keywords:** primary CMV infection, virus-specific cell-mediated immunity, immunocompetent adults, correlates of maternal protective immunity for the fetus

## Abstract

Cytomegalovirus-specific cell-mediated immunity (CMV-CMI) in actively infected healthy immunocompetent hosts has been poorly investigated. Conversely, correlates of maternal protective immunity for the fetus after primary infection in pregnancy continue to be studied. The kinetics and magnitude of CMV-specific CMI in immunocompetent primary CMV-infected adults are described. A literature review on CMV-CMI in primarily infected pregnant women and its correlation to the risk of vertical virus transmission is included. Immunological measurements after infection were performed by enzyme-linked ImmunoSPOT assay enumerating IFN-γ secreting CMV-specific T cells, at a single cell level, upon in vitro stimulation with viral antigens. Simultaneously, serological and virological profiles of infected patients were investigated. Patients displayed mild-to-moderate clinical and laboratory profiles for infection, and all showed positive EliSpot results in the early stage of infection (<20 days after onset). The virus-CMI was strong in the majority of patients (58.8%) in which the lowest CMV-DNAemia levels (<300 copies/mL) were detected. Significantly higher viral loads were observed in patients with weak CMV-CMI at the same time-point post-infection (up to 15,104 copies/mL; *p* < 0.001). T cell response magnitudes to IE-1 and pp65-UL83 peptides were overlapping and stable over time. In these case series, the early presence of CMV-CMI was probably pivotal in controlling viral replication and led to spontaneous viral clearance.

## 1. Introduction

Cytomegalovirus (CMV) is a ubiquitous and highly prevalent human herpes virus, with an estimated worldwide seroprevalence ranging from 45% to 100% [1,2]. In the context of immature or immunocompromised immune systems, such as in utero acquisition or post-transplant acquisition/reactivation, CMV infection is associated with high morbidity and mortality rates [3,4,5]. It is known that virus-specific cell-mediated immunity (CMI) is an essential host factor in the control of viral replication; therefore, studies investigating the utility of immunological measurements in the assessment of congenital CMV disease severity and in the clinical management of post-transplant CMV infection were performed [6,7,8,9]. On the other hand, in the context of immunocompetence, CMV infection is traditionally considered asymptomatic or only causing a mild disease [10], and the virus-specific CMI in the actively infected healthy immunocompetent host has been poorly investigated [11]. Separate mention should be made for pregnant women given that, in recent years, potential maternal immune correlates of protection from CMV transmission to the fetus after primary infection in pregnancy have been studied [12,13,14,15,16,17].

Here, we describe the kinetics and the magnitude of CMV-specific CMI in immunocompetent adults with primary CMV infection. Moreover, a literature review on the CMV-specific CMI in primarily infected pregnant women and its correlation to the risk of vertical virus transmission has been included here.

## 2. Materials and Methods

### 2.1. Study Case Series

Seventeen immunocompetent adult patients with primary and symptomatic CMV infection and whose serological, virological, immunological, and clinical profiles of infection are described. Infection symptoms and signs at the time of presentation are reported in Table 1. 

Diagnosis of primary CMV infection was based on serological findings such as the presence of CMV-specific IgM without detectable CMV-specific IgG (documented seroconversion) or with detectable CMV-specific IgG and IgM associated with low avidity indices of IgG. In all cases, anti-CMV IgM was confirmed using a homemade immunoblot for the detection of CMV-specific IgM [18]. Onset of CMV infection was approximately estimated taking into account when CMV-related symptoms appeared and biochemical/hematological signs. The patients were referred to the IRCCS St. Orsola Polyclinic of Bologna; three-month follow-up was available for all patients, and five patients (29.4%) were followed for 6 months after the onset of infection. Written informed consent was obtained from the patients for the publication of these case reports. Institutional approval was not required to publish the case details.

### 2.2. Serological Profile of Infection

Serological measurements were performed on serum samples by using LIAISON^®^ CMV IgG, IgM and IgG Avidity II assays (DiaSorin S.p.A., Saluggia, Italy) based on the chemiluminescent immunoassay (CLIA) technology. The LIAISON^®^ CMV IgG II assay measures the concentration of anti-CMV IgG in serum or plasma samples and results <12 AU (arbitrary units)/mL are considered negative, and ≥14 AU/mL are positive; values between 12 and 14 AU/mL are considered equivocal. The LIAISON^®^ CMV IgM II assay enables the semi-quantitative determination of anti-CMV IgM in serum or plasma samples, and results <18 AU/mL are considered negative and ≥22 AU/mL are positive; values between 18 and 22 AU/mL are considered equivocal. Finally, the LIAISON^®^ CMV IgG Avidity II assay determines IgG avidity in serum and plasma samples in which CMV-specific IgG is demonstrated; avidity indexes (AIs) of <15%, between 15% and 25%, and >25% indicate low, moderate, and high avidity, respectively. All the assays were performed on the fully automated random-access LIAISON^®^ XL analyzer (DiaSorin S.p.A., Saluggia, Italy), according to the manufacturer’s instructions.

The homemade immunoblot for the detection of CMV-specific IgM was prepared, performed, and interpreted as previously described [19].

### 2.3. Virological Profile of Infection

Assay of CMV-DNA was performed on EDTA-anticoagulated whole blood samples by using a commercial quantitative real-time PCR.

DNA extraction was performed with the QIAsymphony SP instrument (Qiagen, Hilden, Germany) and the quantification of CMV-DNA was performed by using a CMV ELITe MGB^®^ kit (ELITech Group, Turin, Italy) on the ABI Prism 7500 real-time PCR System (PE Applied Biosystem, Foster City, CA, USA). The extraction and amplification procedures were performed as previously described [18]. The analytical sensitivity of the assay is 10 copies of target DNA per amplification reaction. The lower limit of quantification (LLQ) of the assay is 300 copies/mL WB.

### 2.4. Immunological Profile of Infection

Immunological measurements were performed by using an EliSpot assay (EliSpot Interferon-γ Basis Kit; GenID GmbH, Strasburg, France) that enumerates IFN-γ secreting CMV-specific T cells (both CD4+ and CD8+ cells), at a single cell level, upon in vitro stimulation with viral antigens (CMV IE-1 and pp65-UL83 peptides; GenID GmbH, Strasburg, France). A mitogen stimulation and a negative control were included to determine general T cell responsiveness and background, respectively. The assay was performed and interpreted as previously described [20]. Briefly, the EliSpot assay cut-off value for a positive response to viral stimulation was ≥5 spot-forming cells (SFCs)/2 × 10^5^ peripheral blood mononuclear cells. A positive EliSpot result identified a patient with detectable CMV-specific CMI. Regarding the magnitude of the CMV-specific CMI, it was considered weak when the number of SFCs was between 5 and 20, good between 21 and 100, and very good ≥100. The maximum EliSpot value was limited to 500 SFCs/2 × 10^5^ peripheral blood mononuclear cells, because 500 spots represents the saturating concentration of the EliSpot well. The formula “sample—negative control” was applied in all SFC measurements.

Ten CMV-seropositive adults with remote infection (>3 years) and 3 CMV-seronegative healthy immunocompetent adults were investigated by EliSpot assay as a preliminary experiment. In the seropositive patients, the CMV-specific CMI ranged from 18 to 252 SFCs/2 × 10^5^ peripheral blood mononuclear cells.

### 2.5. Statistical Analysis

As non-parametric significance tests, the Mann–Whitney and Kruskal–Wallis tests were performed when appropriate. Positive samples below the LLQ of the DNAemia assay were censored with a value corresponding approximatively to one-half of the LLQ (i.e., 150 copies/mL WB). An arbitrary value of 550 SFCs/2 × 10^5^ peripheral blood mononuclear cells was attributed to the saturated wells.

Statistical analyses were performed using the GraphPad Prism software (San Diego, CA, USA), version 7.04. *p* < 0.05 was considered statistically significant.

## 3. Description of the Case Series

Symptoms and signs, along with abnormal laboratory findings that had aroused physician suspicion of CMV infection, are reported in Table 1.

Patients’ mean age was 35 ± 9.9 years, and 11 (64.7%) were male. Primary Epstein–Barr virus infection was excluded in all cases based on patients’ serological profiles, i.e., the absence of Epstein–Barr virus-specific IgM and the presence of IgG anti-Epstein–Barr virus nuclear antigens. No patients had congenital or acquired immunodeficiency syndrome, history of allogeneic transplant, recent/ongoing immunosuppressive therapy, or chemo/radiotherapy or documented morbidities that could be associated with a degree of immune dysfunction (e.g., diabetes mellitus). Furthermore, the CD4 lymphocyte count was studied in all patients at the time of presentation, and the lowest value was equal to 672 cells/μL (median value, 1057 cells/μL (range, 672–2064)).

Risk factors for CMV infection were household contact with symptomatic (influenza-like-illness) and asymptomatic young children in 41.2% (7/17) and 17.6% (3/17) of cases, respectively. Furthermore, in 23.6% (4/17) of cases, patients worked in a hospital setting. For the remaining 3 patients (17.6%), sexual transmission of the virus was hypothesized.

Patients displayed mild to moderate clinical and laboratory profiles for primary CMV infection (Table 1); none displayed organ disease. In particular, a history of fever was reported by 94.1% (16/17) of patients, followed by headache in 82.3% (14/17) of patients. A slight increase in leukocyte count (median value, 12.72 × 10^9^/L; range 10.80–14.32 × 10^9^/L) was observed in 30% (5/17) of patients, while lymphocytosis was observed in most of the patients (82.3%; 14/17). The most commonly altered biochemical parameter was the hepatic transaminase being elevated: this occurred in 88.2% (15/17) of patients. Normal levels of bilirubin (normal value < 1.2 mg/dL) were observed in all the patients (100%).

Patients were referred to the IRCCS St. Orsola Polyclinic of Bologna after a median time of 9 days (range, 7–15 days) from the onset of symptomatology, and 64.7% of them (11/17 patients) were taking antibiotics prescribed by their own general practitioner; the median duration of antibiotic therapy at home was 5 days (range, 3–8). The serological diagnosis of acute primary CMV infection was made after a median time of 4 days (range, 3–8) since patients were referred to our Centre. CMV infection required hospitalization in 76.4% (13/17) of patients, with a median duration of stay of 5 days (range, 4–10 days).

**Figure 1:**
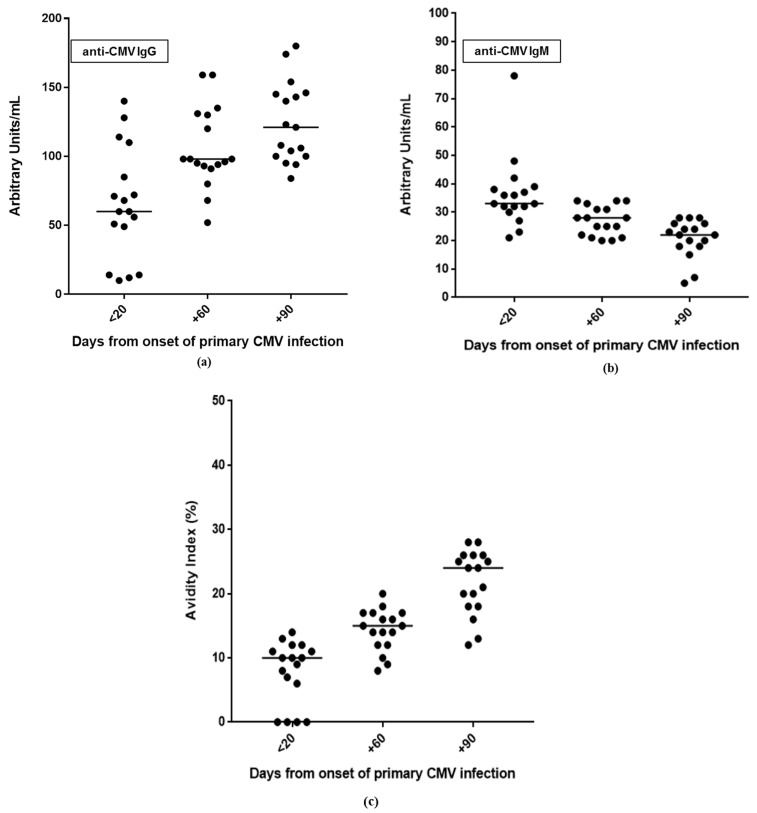
Kinetics of antibody responses during the three-month follow-up after primary CMV infection: (**a**) anti-CMV IgG and (**b**) IgM in serum samples; (**c**) IgG avidity index.

The kinetics of antibody responses during the first year after infection are reported in Figure 1. In particular, anti-CMV IgG was detectable in 76.5% (13/17) of patients within 20 days after the onset of infection, and in all patients (100%) at day +60. IgG avidity index resulted low or moderate in all patients (100%) at day +60, and in 58.8% (10/17) of patients at day +90. Finally, with regard to anti-CMV IgM, positive results were observed in 58.8% (10/17) of patients at day +90 (IgG positive/IgM positive patients); equivocal (*n* = 3) and negative (*n* = 4) results were observed in the remaining 41.2% of patients (7/17).

With regard to the CMV-specific CMI, all patients (100%) had a positive EliSpot result at the first time-point analyzed (i.e., <20 days) (Table 2). In particular, 58.8% (10/17) of patients had a very good CMV-specific CMI; 11.8% (2/17) and 29.4% (5/17) of patients had good and weak virus-specific immune responses, respectively.

**Figure 2:**
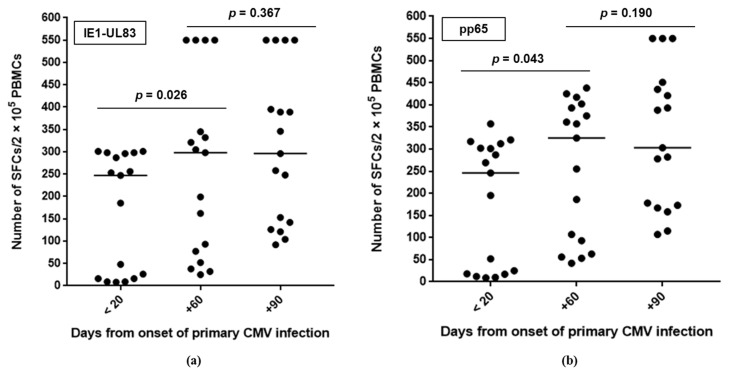
Number of cells reactive with CMV peptides during the three-month follow-up after primary CMV infection: (**a**) pp65-UL83 and (**b**) IE1. The line indicates the median value. SFCs, spot-forming cells; PBMCs, peripheral blood mononuclear cells.

The number of cells which were reactive with CMV pp65-UL83 and IE1 peptides overlapped at each time-point. In particular, CMV-specific CMI increased within the two months after the onset of infection and was stable at the next time-point (Figure 2).

**Figure 3:**
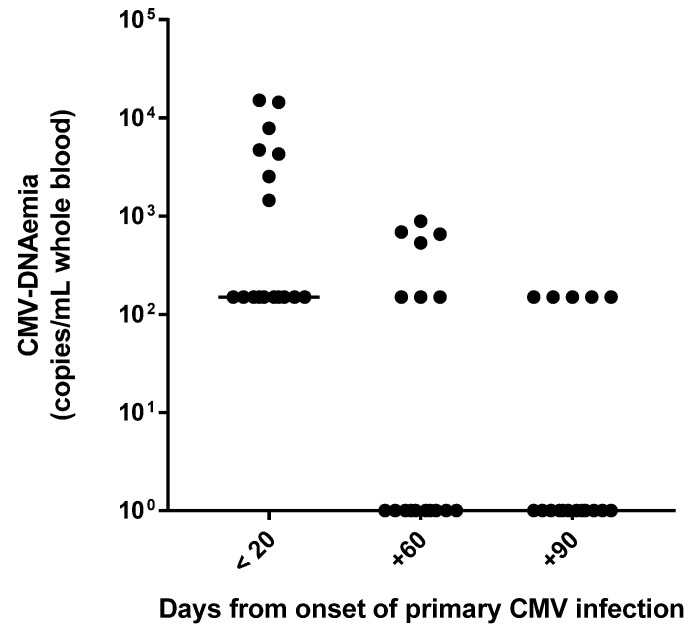
CMV-DNAemia values during the three-month follow-up after primary CMV infection.

By analyzing the CMV-specific CMI in relation to viral replication, it was observed that all patients with a very good CMV-specific CMI within 20 days after primary infection had low positive CMV-DNA results with values below the LLQ (i.e., <300 copies/mL) of the molecular assay. On the other hand, the five patients with weak CMV-specific CMI had a median value of viral load equal to 7853 (range, 4727–15,104) copies/mL (*p* < 0.001). Overall, in the majority of cases (12/17; 70.6%), CMV-DNA negativity was observed at day +90 (Figure 3). The remaining five patients (29.4%) underwent further virological and immunological controls after 3 months (at day +180), and two of them still showed a positive CMV DNAemia result (i.e., <300 copies/mL). In general, in all patients, the magnitude of CMV-specific CMI observed at day +180 overlapped with that observed at day +90, suggesting that virus-specific immunity is stable over time. In this regard, all 10 healthy subjects with a remote infection showed a CMV-specific CMI, ranging from weak to very good. As expected, all the CMV-seronegative healthy subjects had a negative EliSpot result.

The resolution of symptomatology occurred after a median time of 5 weeks (range, 1–15) from the onset of infection. Asthenia/malaise was the symptom with the longest duration. In this series of patients, none received antiviral therapy and no complications from primary CMV were observed.

## 4. Discussion

Symptomatic primary CMV infection, in terms of clinical and laboratory profile, in immunocompetent adults without organ specific CMV disease, is currently poorly characterized [10]. Similarly, limited amounts of data in the literature on virus-specific cell-mediated immune responses in non-pregnant immunocompetent adults with symptomatic primary CMV infection are available [11]. The present case series contributes to more complete knowledge about these two topics. As expected, young children were identified as the source of CMV for more than half (58.8%) of the patients [21], and in these cases, the household contact with children referred by patients helped to direct the diagnosis.

Almost all patients displayed general symptoms such as fever (94.1%) and headache (82.3%), and the laboratory profile for CMV infection was also relatively non-specific (the most frequent finding was abnormal liver function test results). These findings support what has been previously reported by other authors, i.e., CMV infection poses a significant diagnostic challenge in immunocompetent adults [1]. Moreover, when referred to our Centre, more than half of patients (64.7%) were taking unnecessary antibiotics prescribed by their own general practitioner, suggesting that, currently, there is a low index of clinical suspicion for CMV infection at initial presentation [1].

The evaluation of antibody responses and CMV-specific CMI during the first year after onset of infection showed that a robust immune response is generated soon after infection. Anti-CMV IgG was undetectable in only 23.5% of patients within 20 days after the onset of infection, and 41.2% of patients had a high IgG avidity index at day +90. However, a persistent anti-CMV IgM antibody, defined as stable IgM values for ≥3 months [22], was observed in 58.8% (10/17) of patients.

With regard to virus-specific CMI, all patients in the early stage of infection, i.e., <20 days after onset, showed a positive EliSpot result (to both IE-1 and pp65-UL83 peptides), and in the majority of cases (58.8%), the virus-specific CMI was strong. These findings are in line with data reported in the literature [12,13,14,23], although immunocompetent pregnant women with primary CMV infection were mostly studied. However, it was shown that although the CMV-specific T cell response was detected early after primary infection (already 15 days after the onset of infection), some effector functions were acquired late by T cells [12,13,24]. Regarding the frequencies of protein-specific T cells (both CD4+ and CD8+), we observed an overlapping magnitude of T cell responses to IE-1 and pp65-UL83 peptides at each time-point. Recently, Mele and colleagues analyzed the frequencies of CD4+ and CD8+ T cells specific for four CMV proteins, IE-1, pp65, gHgLpUL128L (pentamer) and gB, in the memory pool of six immunocompetent patients with primary CMV infection (within one month and at 6–12 months after infection onset (at early and late stages of infection, respectively)) [14]. In particular, the evaluation of the frequencies of protein-specific CD4+ and CD8+ memory T cells in the immunocompetent patients with primary CMV infection showed that proteins gB and pp65 were immunodominant targets of memory CD4+ T cells, and IE-1 protein and pp65 were immunodominant targets of CD8+ T cells; the pattern of T cell reactivity was comparable at early and late stages of infection.

In our case series, the early presence of virus-specific CMI was probably pivotal in controlling viral replication. Indeed, as previously reported [14,25], the CMV load detected in the blood of patients was low. Notably, the lowest CMV DNAemia levels were detected in patients with a very good CMV-specific CMI within 20 days after the onset of infection (all CMV-DNA values were <LLQ of the molecular assay). On the contrary, significantly higher CMV DNAemia values were observed in patients with a weak CMV-specific CMI (up to 15,104 copies/mL) at the early stage of infection (*p* < 0.001). However, all patients had a good/very good viral-specific CMI after two months post-onset of infection, which probably led to the spontaneous clearance of infection without antiviral treatment administration. In a recent study involving 20 immunocompetent adults who developed symptomatic severe CMV primary infection despite effective innate and adaptive immune responses, Riou and colleagues hypothesized a possible role of the CMI in the pathogenesis of tissue damage due to both massive lymphocytosis and excessive lymphocyte activation; however, the authors did not exclude possible immune evasion mechanisms at the early stage of infection employed by CMV strains [11].

Given that the in utero acquisition of CMV infection constitutes a major public health concern, parameters of the T cell immune response to primary CMV infection have recently been investigated in pregnant women [26]. Studies aimed to determine whether immune responses are functionally impaired during pregnancy. In particular, some authors found no difference between pregnant and non-pregnant individuals, either at the qualitative or quantitative level of CMV-specific immune response [12,15], although others observed a functional exhaustion of CMV-specific CD4+ T cells in pregnant women with primary CMV infection [27]. Moreover, some studies have investigated functional and phenotypic immunological features by comparing women who did not transmit and those who had transmitted infection to the fetus, with the aim of defining reliable correlates of maternal immune protection from vertical virus transmission. Of note, although it is known that virus-specific CMI is essential in the viral replication control and is associated with less severe CMV disease in the immunocompetent host, discordant data are so far reported in the literature on the topic of maternal immunity roles in vertical virus transmission. Specifically, some authors have showed that the development of a T cell response to primary CMV infection, particularly CD4+ T cells, seems to be crucial for the control of CMV vertical transmission [12,13,14]. In particular, Lilleri et al. found a significantly more rapid development of the CD4+ T cell lymphoproliferative response in non-transmitting women compared to transmitting women [12], confirming previous observations by Revello et al. [15]. Fornara et al. found a significantly higher IL-2 production by CMV-specific CD4+ T cells (at 30 days post-primary infection) and a significantly higher frequency of both specific CD4+ and CD8+ CD45RA+ effector memory T cells (at 60 days post-primary infection) in non-transmitting than in transmitting women [13]. Finally, Mele et al. observed a significantly higher frequency of CMV-specific CD4+ T cells with a long-term IL-7R^pos^ memory phenotype in non-transmitting compared to transmitting women [14]. A critical role for maternal CD4+ T cells was also demonstrated by Bialas et al., who used a Rhesus animal model of congenital CMV transmission; indeed, the authors found that the presence of maternal CD4+ T cell immunity was important for inducing protective immune responses which prevented severe CMV-associated fetal disease [28]. On the contrary, some authors have observed an association between low CMV-specific IFN-γ+ T cells and non-transmission to fetuses [16,18]. It could be speculated that a stronger activation of the CMI is due to a less efficient viral clearance, leading to higher risk of transmission [29]. Finally, Mele et al. found that the pattern of T cell specific reactivity (both the individual CD4+ or CD8+ T cell frequencies specific for IE-1, pp65, gHgLpUL128L, gB and the sum of the frequencies for all the four viral proteins) was not significantly different between transmitting and non-transmitting women [14].

However, comparison of the studies’ results is limited due to differences in assay techniques and infection time-points analyzed.

## 5. Conclusions

This case series showed that in primarily infected immunocompetent adults, the presence of CMV-specific CMI at the early stage of infection and which is stable over time is indicative of a favorable clinical prognosis.

The magnitude of CMV-specific CMI is associated with virological and clinical profiles of infection, implying that immunological, combined with virological measurements, can provide useful adjunctive information on the clinical course of infection, contributing toward personalization of the clinical management.

Finally, further studies on maternal immunological parameters are needed in order to reliably differentiate, at the individual level, which mothers with primary CMV infection will transmit the virus to the fetus. Studies in this field are also crucial for progress toward the development of both effective antiviral treatments during pregnancy, and vaccines in order to prevent congenital CMV transmission.

## Figures and Tables

**Table 1:** Clinical and laboratory profile for primary CMV infection at the time of presentation.

Symptoms	No. of Patients (%)
Headache	14 (82.3)
Asthenia/malaise	9 (52.9)
Arthralgia/myalgia	6 (35.3)
Gastrointestinal disorders (nausea/vomiting/diarrhea/abdominal pain)	4 (23.5)
**Signs**	
Fever > 38 °C	11 (64.7)
Fever < 38 °C	5 (29.4)
Hepatosplenomegaly	9 (52.9)
Lymphadenomegaly	9 (52.9)
Rash	2 (11.8)
**Abnormal laboratory finding**	
Elevated total white cell count (reference range, 3.60–10.50 × 10^9^/L)	5 (30)
Neutrophil-to-lymphocyte ratio < 1	14 (82.3)
CD4+/CD8+ ratio < 1	10 (58.8)
Lymphocyte activation	11 (64.7)
High erythrocytes sedimentation rate (reference range, <38.0 mm/h)	9 (52.9)
High levels of c-reactive protein (VN, <5 mg/L)	12 (70.6)
Elevated sGPT (NV, <40 U/L)	15 (88.2)
sGPT > 2–5 NV	7 (41.2)
sGPT > 5–10 NV	7 (41.2)
sGPT > 10 NV	4 (23.5)
Elevated LDH levels (reference range, 135–225 U/L)	13 (76.4)
Elevated ALP (reference range, 40–129 U/L)	7 (41.2)
Elevated GGT (reference range, 8–61 unit/L)	9 (52.9)

sGPT, serum glutamic pyruvic transaminase; NV, normal value; LDH, lactate dehydrogenase; ALP, alkaline phosphatase; GGT, gamma glutamyl transferase.

**Table 2:** Immunological, virological, and clinical findings in primary CMV infection within 20 days after the onset of infection.

Pt	CMV-Specific CMI ^1^	CMV-DNAemia Copies/mL WB	Signs and Symptoms
2	Very good	Positive < 300 ^2^	Headache, lymphadenomegaly
3	Very good	Positive < 300	Fever < 38 °C, headache
4	Very good	Positive < 300	Fever < 38 °C, rash
6	Very good	Positive < 300	Fever < 38 °C, asthenia/malaise
8	Very good	Positive < 300	Fever < 38 °C, headache, gastrointestinal disorders
16	Very good	Positive < 300	Fever > 38 °C, headache, hepatosplenomegaly
10	Very good	Positive < 300	Fever > 38 °C, headache, lymphadenomegaly
11	Very good	Positive < 300	Headache, rash, lymphadenomegaly
13	Very good	Positive < 300	Fever < 38 °C, headache, arthralgia/myalgia, hepatosplenomegaly
17	Very good	Positive < 300	Hepatosplenomegaly, asthenia/malaise lymphadenomegaly
1	Good	1450	Fever > 38 °C, headache, gastrointestinal disorders, asthenia/malaise, hepatosplenomegaly, lymphadenomegaly
14	Good	2536	Fever > 38 °C, headache, asthenia/malaise, hepatosplenomegaly, lymphadenomegaly
5	Weak	7853	Fever > 38 °C, headache, arthralgia/myalgia, hepatosplenomegaly
7	Weak	15,104	Fever > 38 °C, headache, asthenia/malaise, gastrointestinal disorders
12	Weak	14,423	Fever > 38 °C, headache, asthenia/malaise, arthralgia/myalgia hepatosplenomegaly, lymphadenomegaly
15	Weak	4727	Fever > 38 °C, headache, asthenia/malaise, arthralgia/myalgia, hepatosplenomegaly, lymphadenomegaly
9	Weak	4295	Fever > 38 °C, headache, asthenia/malaise, arthralgia/myalgia, hepatosplenomegaly, lymphadenomegaly, gastrointestinal disorders

Pt, patient; CMI, cell-mediated immunity; WB, whole blood. ^1^ CD8+ and CD4+ T cell responses to both CMV pp65 and IE1 antigens were taken into account; ^2^ Lower limit of quantification of the molecular assay.

## Data Availability

Data is contained within the article.
